# Caspase polymorphisms and prognosis of hepatocellular carcinoma

**DOI:** 10.1371/journal.pone.0176802

**Published:** 2017-04-28

**Authors:** Song Zhang, Qianyi Xiao, Zhuqing Shi, Guopeng Yu, Xiao-Pin Ma, Haitao Chen, Pengyin Zhang, Suqin Shen, He-Xi Ge Sai-Yin, Tao-Yang Chen, Pei-Xin Lu, Neng-Jin Wang, Weihua Ren, Peng Huang, Jun Xie, Carly Conran, S. Lilly Zheng, Long Yu, Jianfeng Xu, De-Ke Jiang

**Affiliations:** 1 State Key Laboratory of Genetic Engineering, Collaborative Innovation Center for Genetics and Development, School of Life Sciences, Fudan University, Shanghai, China; 2 Ministry of Education Key Laboratory of Contemporary Anthropology, School of Life Sciences, Fudan University, Shanghai, China; 3 Center for genetic Epidemiology, School of Life Sciences, Fudan University, Shanghai, China; 4 Center for Genetic Translational Medicine and Prevention, School of Public Health, Fudan University, Shanghai, China; 5 Department of Urology, The 9th People’s Hospital of Shanghai, School of Medicine, Shanghai Jiaotong University, Shanghai, China; 6 Qidong Liver Cancer Institute, Qidong People’s Hospital, Qidong, Jiangsu, China; 7 Central Laboratory, Luoyang Central Hospital Affiliated to Zhengzhou University, Luoyang, Henan, China; 8 College of Basic Medicine, Shanghai University of Medicine and Health Sciences, Shanghai, China; 9 Program for Personalized Cancer Care, NorthShore University HealthSystem, Pritzker School of Medicine, University of Chicago, Evanston, Illinois, United States of America; 10 Fudan Institute of Urology, Huashan Hospital, Fudan University, Shanghai, China; 11 State Key Laboratory of Organ Failure Research, Guangdong Key Laboratory of Viral Hepatitis Research, Department of Infectious Diseases and Hepatology Unit, Nanfang Hospital, Southern Medical University, GuangZhou, China; University of North Carolina at Chapel Hill School of Medicine, UNITED STATES

## Abstract

The aim of our study was to determine the impact of genetic polymorphisms in the caspase (CASP) genes on prognosis of hepatocellular carcinoma (HCC). We genotyped 7 potentially functional polymorphisms in *CASP3*, *CASP7*, *CASP8*, *CASP9*, *CASP10* genes in 362 HCC patients of receiving surgical resection of HCC tumor. The associations of genotype and haplotype with overall survival (OS) and disease free survival (DFS) were analyzed by using the Cox proportional hazards model. We found that the *CASP9* rs4645981 C allele was significantly associated with positive effect on DFS (*P* = 0.011 and 0.016 for CT+CC vs. TT in univariate and multivariate analysis, respectively), CT genotype was associated with a better OS of HCC than the TT genotype both in univariate and multivariate analysis (*P* = 0.048 and 0.041, respectively). Moreover, the *CASP3* rs2705897 GT genotype showed marginally significant association with decreased OS and DFS, compared with the GG genotype. One haplotype TT/TG in *CASP3* (constructed by rs12108497 T>C and rs2705897 T>G) was significantly associated with decreased OS and DFS, compared to the common haplotype TT/TT both in univariate analysis (*P* = 0.021 and 0.026, respectively) and multivariate analysis (*P* = 0.025 and 0.030, respectively). The haplotype GT/GT in *CASP9* (constructed by rs4645978 A>G and rs4645981 C>T) was significantly associated with decreased DFS both in univariate and multivariate analysis (*P* = 0.012 and 0.010, respectively). In conclusion, the *CASP9* rs4645981 polymorphism, *CASP3* and *CASP9* haplotypes may be useful prognosis markers for HCC patients with surgical resection of tumor.

## Introduction

Hepatocellular carcinoma (HCC), mainly caused by chronic hepatitis B virus (HBV) and hepatitis C virus (HCV) infections, is one of the most commonly diagnosed cancers and the third leading cause of cancer-related death worldwide [1]. In 2012, about 782,500 new HCC cases and 745,500 deaths occurred in the world, making the incidence and mortality rates almost equal [2]. Although attempts have been made to predict recurrence and prognosis in HCC patients using clinical factors, such as positive portal vein thrombosis, large tumor size, increased serum alpha-fetoprotein (AFP), vascular invasion and advanced tumor node metastasis (TNM) stage, the long-term prognosis remains poor with reported 5-year survival rates ranging from 17% to 53% [3]. Therefore it is essential to better understand the mechanism of cancer progression and development in HCC and identify potential biomarkers for prognosis prediction.

Apoptosis is a genetically controlled process of cell suicide, which plays an important role in multicellular organisms [4]. As we all know, inappropriate regulation of apoptosis mechanism facilitates the accumulation of somatic mutations and thereby contributes to tumor initiation, progression as well as metastasis [5–6]. Caspases (CASPs), members of a conserved family involved in signaling and execution in apoptosis pathways, are cysteine-aspartic acid proteases, which can be broadly divided into initiator (upstream) and effector (downstream) CASPs based on their functions [7]. To date, 14 family members have been identified [8]. *CASP3*, *CASP6*, and *CASP7* belong to effector CASPs and they execute cell death process; *CASP8*, *CASP9*, and *CASP10* transmit apoptotic signals and they belong to initiator CASPs [9]. All known CASPs possess an active-site cysteine and they cleave their substrates after the aspartic acid residue [10].

Many studies have shown that genetic polymorphisms in CASP genes are associated with risk of various human cancers, including HCC [9, 11–15]. However, the influence of the CASP genes-related polymorphisms on the prognosis of HCC have not been investigated extensively. Therefore, we selected 7 potentially functional SNPs in *CASP3*, *CASP7*, *CASP8*, *CASP9*, and *CASP10* genes and aimed to determine whether polymorphisms in these genes are associated with prognosis of HCC.

## Materials and methods

### Patients

From April 1996 to September 2009, a total of 362 Chinese Han patients with primary HCC newly diagnosed and received surgical resection of HCC tumor were recruited by the Qidong Liver Cancer institute in Qidong, Jiangsu province, China. The clinical outcomes of HCC were recorded until October 2014, with a median follow-up time of 53.0 months, which range from 2 to 110 months. The clinical diagnosis of HCC was based on the National Comprehensive Cancer Network (NCCN) clinical practice guidelines in oncology and histopathological examination. Patients with secondary liver cancers were excluded from our study. Patients with no other cancers were determined at the initial screening examination and were followed-up every 3months by researchers from the time of enrollment, stopping until death or the last time of follow-up.

There were no restrictions on gender, age and tumor stage for recruitment and 5 ml whole blood was extracted for each subject. Clinical characteristics such as tumor size, differentiation, venous invasion, and son on were collected via medical records with approval of patients. The clinical typing of tumors were determined by the TNM classification system of International Union Against Cancer (edition 6) and the histologic grade of tumor differentiation was assigned by the Edmondson grading system. Overall survival (OS) and disease free survival (DFS) were used as endpoints for the study. OS was calculated from the date of pathologic diagnosis/recruitment to death or the end of available follow-up.

Disease free survival (DFS) was defined as the time from pathologic diagnosis/recruitment to disease recurrence, metastasis, disease specific death or last follow-up.

Written informed consent was obtained from each patient before enrollment, and this study was approved by the Department of Scientific Research of Fudan University as well as the Qidong Liver Cancer Institute.

### SNP selection

To select potentially functional SNPs in *CASP3*, *CASP7*, *CASP8*, *CASP9*, and *CASP10* genes, we utilized the International HapMap Project database (http://hapmap.ncbi.nlm.nih.gov/), and the dbSNP database (https://www.ncbi.nlm.nih.gov/projects/SNP/). Finally, a total of 7 SNPs were selected for genotyping (Table 1).

**Table 1:** SNPs selected in CASP genes and their allele frequencies.

Gene	Chromosome	Location	Position	SNP	Allele	MAF (CHB)^a^	MAF (observed)^b^
***CASP3***	4q34	5' flank	185571557	rs12108497	T>C	0.282	0.256
		5' flank	185553098	rs2705897	A>C	0.209	0.162
***CASP7***	10q25	T244S	115489152	rs2227310	C>G	0.427	0.391
***CASP8***	2q33-q34	Intron	202151163	rs3769818	G>A	0.291	0.265
***CASP9***	1p36.21	5' flank	15852034	rs4645978	A>G	0.379	0.368
		5' flank	15851483	rs4645981	C>T	0.214	0.136
***CASP10***	2q33-q34	L522I	202082459	rs13006529	T>A	0.185	0.184

MAF, minor allele frequency; CHB, Chinese Han in Beijing; SNP, single nucleotide polymorphism.

^a^ MAF in Chinese Han population in Hapmap database.

^b^ MAF in our studied population.

### DNA extraction and genotyping

Genomic DNA was extracted from blood samples using the QIAamp DNA Mini Kit (GIAGEN GmbH, Hilden, Germany). Genotyping was performed with Sequenom MassARRAY iPLEX platform by use of allele-specific MALDI-TOF mass spectrometry assay. Polymerase chain reaction (PCR) and extension primers for these 7 SNPs were designed using the MassARRAY Assay Design 3.0 software (Sequenom). Duplicate test samples and two water samples (PCR negative controls) were included in each 96-well plate. Genotyping quality was examined by a detailed QC procedure consisting of >95% successful call rate, duplicate calling of genotypes, internal positive control samples.

### Statistical analysis

The haplotypes were constructed for the genes with at least two SNPs using Bayesian algorithm by PHASE software. Survival curves were estimated using the Kaplan-Meier method. Hazard ratios (HRs) and 95% confidence intervals (CIs) were estimated for each analysis by using the Cox proportional hazards regression model. The effects of clinical variables, single SNP and haplotype on OS and DFS were assessed using the Cox proportional hazards regression model and log-rank test. All analyses were performed with SPSS software version 22 (SPSS, Chicago, IL). All tests were two-sided and a *P*<0.05 was considered statistically significant.

## Results

### Patient characteristics and clinical predictors

The clinical pathologic characteristics of the 362 HCC patients and their associations with OS are summarized in Table 2. There were 225 (62.2%) deaths at the time of analysis and the overall median survival time (MST) was 34.0 (95%CI, 27.4–40.6) months. In univariate analysis, tumor size and venous invasion were significantly associated with OS (*P* = 0.028 and 0.029, respectively) and DFS (*P* = 0.042 and 0.026, respectively). However, none of other clinical characteristics was significantly associated with OS or DFS.

**Table 2:** Clinical characteristics and their prediction of overall survival and disease free survival in HCC patients.

**Characteristics**	No of patients	No of events	5-y-survival (%)	MST (95%CI)	Overall survival (OS)			Disease free survival (DFS)		
					Log-rank *P*	Hazard ratio (95% CI)	*P*	Log-rank *P*	Hazard ratio (95% CI)	*P*
**Number**	362	225	30	34.0 (27.4–40.6)						
**Age (year)**					0.478			0.410		
≤50	186	113	30	35.0 (23.3–46.7)		1.00				
>50	176	112	29	33.0 (24.2–41.8)		1.10 (0.85–1.43)	0.483		1.11 (0.86–1.45)	0.417
**Sex**					0.476			0.665		
female	63	41	27	31.0 (24.2–37.8)		1.00				
male	299	184	30	37.0 (27.5–46.5)		0.89 (0.63–1.24)	0.481		0.93 (0.66–1.30)	0.670
**Smoking**					0.265			0.200		
never	224	144	26	31.0 (23.2–38.8)		1.000				
ever	138	81	37	39.0 (27.3–50.7)		0.86 (0.65–1.13)	0.270		0.84 (0.64–1.10)	0.207
**Drinking**					0.615			0.728		
never	142	86	28	35.0 (19.6–50.4)		1.00				
ever	220	139	31	33.0 (25.0–41.0)		1.07 (0.82–1.40)	0.619		1.05 (0.80–1.37)	0.731
**Family history**					0.257			0.299		
absent	263	158	31	37.0 (27.3–46.7)		1.00				
present	81	55	25	29.0 (17.4–40.6)		1.19 (0.88–1.62)	0.262		1.17 (0.86–1.60)	0.306
unkown	18	18								
**HbsAg**					0.599			0.478		
negative	59	40	35	22.0 (6.7–37.3)		1.00				
positive	303	185	28	37.0 (30.2–43.8)		0.91 (0.65–1.29)	0.603		0.89 (0.63–1.25)	0.484
**AFP**					0.395			0.266		
negative	142	95	26	33.0 (25.7–40.3)		1.00				
positive	214	127	32	35.0 (26.1–43.9)		0.89 (0.68–1.16)	0.400		0.86 (0.66–1.12)	0.273
unkown	6	3								
**Tumor size (cm)**					0.026			0.039		
≤5	183	107	35	39.0 (28.1–49.9)		1.00				
>5	179	118	24	30.0 (21.0–39.0)		1.34 (1.03–1.75)	0.028		1.31 (1.01–1.71)	0.042
**Differentiation**					0.568			0.390		
Ⅰ+Ⅱ	196	122	28	37.0 (27.5–46.5)		1.00				
Ⅲ+Ⅳ	155	96	32	34.0 (26.0–42.0)		0.93 (0.71–1.21)	0.572		0.89 (0.68–1.16)	0.397
unkown	11	7								
**Tumor capsule**					0.495			0.432		
absent	177	113	28	31.0 (22.7–39.3)		1.00				
present	181	110	31	37.0 (26.1–47.9)		0.91 (0.70–1.19)	0.499		0.90 (0.69–1.17)	0.439
unkown	4	2								
**Venous invasion**					0.026			0.023		
absent	257	150	33	39.0 (29.3–48.7)		1.00				
present	102	73	22	26.0 (20.1–31.9)		1.37 (1.03–1.81)	0.029		1.38 (1.04–1.82)	0.026
unkown	3	2								
**Cirrhosis**					0.706			0.705		
absent	121	79	30	27.0 (13.6–40.4)		1.00				
present	239	145	30	36.0 (29.6–42.4)		0.95 (0.72–1.25)	0.708		0.95 (0.72–1.25)	0.709
unkown	2	1								
**Tumor number**					0.701			0.644		
solitary	279	172	30	34.0 (26.0–42.0)		1.00				
multiple	83	53	27	35.0 (24.5–45.5)		1.06 (0.78–1.45)	0.704		1.07 (0.79–1.46_	0.649
**pTNM stage**					0.225			0.339		
Ⅰ+Ⅱ	309	188	31	37.0 (30.7–43.3)		1.00				
Ⅲ+Ⅳ	39	27	24	22.0 (13.0–31.0)		1.28 (0.86–1.92)	0.231		1.21 (0.81–1.82)	0.346
unkown	14	10								

MST, median survival time; CI, confidence interval; AFP, serum α-fetoprotein.

### Association analysis of SNPs with OS and DFS of HCC patients

**Fig 1:**
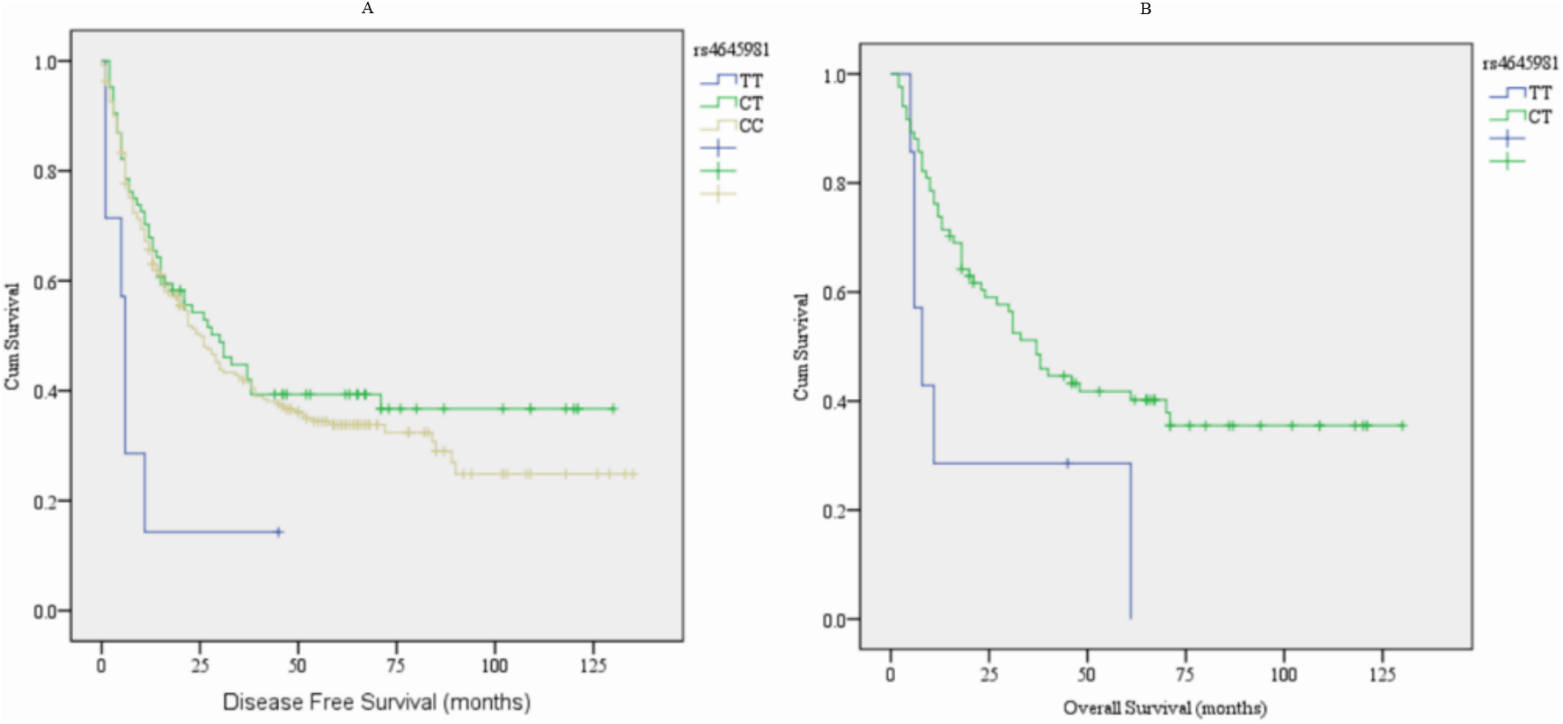
Kaplan-Meier survival curves of *CASP9* rs4645981 with clinical outcomes of 362 HCC patients. (A) disease free survival (DFS) (B) overall survival (OS).

There are 7 SNPs among CASP genes (*CASP3*, *CASP7*, *CASP8*, *CASP9*, *CASP10*) in the present study. The associations of these genetic polymorphisms with OS and DFS are detailed in Table 3. In univariate analysis, the *CASP3* rs2705897 GT genotype possessed a marginally significant association with decreased OS and DFS, compared with the GG genotype (*P* = 0.072 and 0.078, respectively, Table 3). The *CASP9* rs4645981 CT, CC and CT+CC genotypes were associated with significantly increased DFS, compared with the TT genotype (*P* = 0.012, 0.013 and 0.011, respectively, Table 3, Fig 1A). However, the *CASP9* rs4645981 CC and CT+CC genotypes showed a marginally significant association with positive effect on OS, compared with the TT genotype (*P* = 0.066 and 0.057, respectively). Furthermore, the rs4645981 CT genotype showed a statistically significant association with OS, compared with the TT genotype (*P* = 0.048, Table 3, Fig 1B).

**Table 3:** Univariate and multivariate Cox regression analysis of genotypes in HCC patients.

**Genotype**	**No of patients**	No of events	5-y-survival	MST (95%CI)	OS				DFS			
					Univariate analysis		Multivariate analysis		Univariate analysis		Multivariate analysis	
					Hazard ratio (95% CI)	*P*	Hazard ratio (95% CI)	*p*^a^	Hazard ratio (95% CI)	*P*	Hazard ratio (95% CI)	*p*^a^
**CASP3_rs12108497**												
**CC**	30	18	46	50.0 (8.0–92.0)	1.0		1.0					
**CT**	125	81	36	38.0 (22.5–53.5)	1.32 (0.79–2.20)	0.288	1.35 (0.81–2.25)	0.254	1.45 (0.87–2.43)	0.155	1.49 (0.89–2.49)	0.129
**TT**	206	126	36	31.0 (22.3–39.7)	1.23 (0.75–2.02)	0.404	1.24 (0.76–2.04)	0.388	1.30 (0.79–2.13)	0.302	1.32 (0.80–2.16)	0.274
**CT+TT**	331	207	36	33.0 (26.8–39.2)	1.26 (0.78–2.04)	0.344	1.28 (0.79–2.07)	0.315	1.34 (0.83–2.17)	0.236	1.37 (0.84–2.22)	0.204
**CASP3_rs2705897**												
**GG**	10	5	47	42.0	1.0		1.0		1.0		1.0	
**GT**	96	69	32	27.0 (17.7–36.3)	2.35 (0.93–5.96)	0.072	2.32 (0.92–5.87)	0.075	2.30 (0.91–5.82)	0.078	2.35 (0.93–5.94)	0.070
**TT**	253	151	38	37.0 (30.3–43.7)	1.49 (0.61–3.64)	0.380	1.63 (0.67–3.99)	0.283	1.52 (0.62–3.71)	0.357	1.68 (0.68–4.10)	0.259
**GT+TT**	349	220	36	33.0 (26.8–39.2)	1.67 (0.69–4.06)	0.259	1.77 (0.73–4.31)	0.207	1.69 (0.70–4.12)	0.245	1.83 (0.75–4.46)	0.182
***CASP7*_rs2227310**												
**CC**	139	87	36	33.0 (21.0–45.0)	1.0		1.0		1.0		1.0	
**CG**	158	95	39	35.0 (27.8–42.2)	0.97 (0.72–1.30)	0.832	0.91 (0.68–1.22)	0.524	0.98 (0.73–1.31)	0.868	0.93 (0.69–1.25)	0.616
**GG**	61	40	33	37.0 (17.5–56.5)	0.98 (0.68–1.43)	0.929	1.01 (0.69–1.48)	0.954	1.03 (0.71–1.50)	0.876	1.06 (0.73–1.55)	0.751
**CG+GG**	219	135	37	37.0 (30.5–43.5)	0.97 (0.74–1.27)	0.844	0.94 (0.72–1.24)	0.669	1.00 (0.76–1.30)	0.960	0.97 (0.74–1.28)	0.828
***CASP8*_rs3769818**												
**CC**	198	122	38	38.0 (25.7–50.3)	1.0		1.0		1.0		1.0	
**CT**	123	76	34	29.0 (18.5–39.5)	1.08 (0.81–1.43)	0.619	1.08 (0.81–1.44)	0.620	1.05 (0.79–1.40)	0.740	1.05 (0.79–1.40)	0.734
**TT**	32	23	33	33.0 (26.5–39.5)	1.18 (0.76–1.84)	0.466	1.16 (0.74–1.81)	0.525	1.24 (0.80–1.94)	0.340	1.22 (0.78–1.92)	0.383
**CT+TT**	155	99	32	31.0 (22.2–39.8)	1.10 (0.84–1.43)	0.495	1.10 (0.84–1.43)	0.514	1.09 (0.84–1.42)	0.528	1.09 (0.83–1.42)	0.546
***CASP9*_rs4645978**												
**AA**	145	93	35	37.0 (29.0–45.0)	1.0		1.0		1.0		1.0	
**AG**	165	99	39	31.0 (18.5–43.5)	0.98 (0.74–1.30)	0.885	0.98 (0.74–1.30)	0.890	1.00 (0.75–0.33)	0.995	1.00 (0.75–1.33)	0.982
**GG**	50	32	34	24.0 (8.0–40.0)	1.11 (0.74–1.66)	0.620	1.11 (0.74–1.66)	0.609	1.12 (0.75–1.67)	0.584	1.11 (0.74–1.65)	0.626
**AG+GG**	215	131	36	31.0 (22.3–39.7)	1.01 (0.77–1.32)	0.952	1.01 (0.77–1.32)	0.953	1.03 (0.79–1.34)	0.854	1.02 (0.78–1.33)	0.883
***CASP9*_rs4645981**												
**TT**	7	6	23	8.0 (2.9–13.1)	1.0		1.0		1.0		1.0	
**CT**	84	50	41	37.0 (27.7–46.3)	0.42 (0.18–0.99)	0.048	0.38 (0.15–0.96)	0.041	0.33 (0.14–0.79)	0.012	0.34 (0.14–0.83)	0.018
**CC**	270	168	36	35.0 (27.0–43.0)	0.47 (0.21–1.05)	0.066	0.51 (0.22–1.17)	0.112	0.35 (0.16–0.80)	0.013	0.37 (0.16–0.86)	0.021
**CT+CC**	354	218	37	35.0 (28.5–41.5)	0.45 (0.20–1.02)	0.057	0.47 (0.21–1.08)	0.077	0.35 (0.15–0.78)	0.011	0.36 (0.16–0.83)	0.016
***CASP10*_rs13006529**												
**AA**	12	8	27	42.0 (3.5–80.5)	1.0		1.0		1.0		1.0	
**AT**	109	62	40	31.0 (17.8–44.2)	0.89 (0.43–1.87)	0.764	0.95 (0.45–2.01)	0.895	0.96 (0.46–2.00)	0.912	1.02 (0.49–2.16)	0.951
**TT**	236	150	37	34.0 (27.1–40.9)	0.92 (0.45–1.87)	0.813	1.02 (0.50–2.09)	0.960	1.04 (0.51–2.12)	0.918	1.20 (0.58–2.47)	0.625
**AT+TT**	345	212	36	34.0 (27.4–40.6)	0.91 (0.45–1.84)	0.789	0.99 (0.49–2.03)	0.987	1.01 (0.50–2.05)	0.977	1.13 (0.56–2.31)	0.733

MST, median survival time; CI, confidence interval.

^a^ Adjusted by tumor size and venous invasion.

A multivariate analysis of genotype association with OS and DFS of HCC patients was conducted by using Cox proportional hazards model, adjusted for the two significant clinical predictors (tumor size and venous invasion), and the results were similar to the univariate analysis. The GT genotype of *CASP3* rs2705897 showed a suggestively negative effect on OS and DFS of HCC patients, compared with the GG genotype (*P* = 0.075 and 0.070, respectively, Table 3). The *CASP9* rs4645981 CT, CC and CT+CC genotypes were still significantly associated with increased DFS, compared with the TT genotype (*P* = 0.018, 0.021 and 0.016, respectively, Table 3). However, the CT+CC genotype of *CASP9* rs4645981 presented a suggestively positive effect on OS, and the CT genotype of rs4645981 showed a positive effect on OS of HCC patients, compared with the TT genotype (*P* = 0.041, Table 3).

### Association analysis of haplotypes with OS and DFS of HCC patients

**Fig 2:**
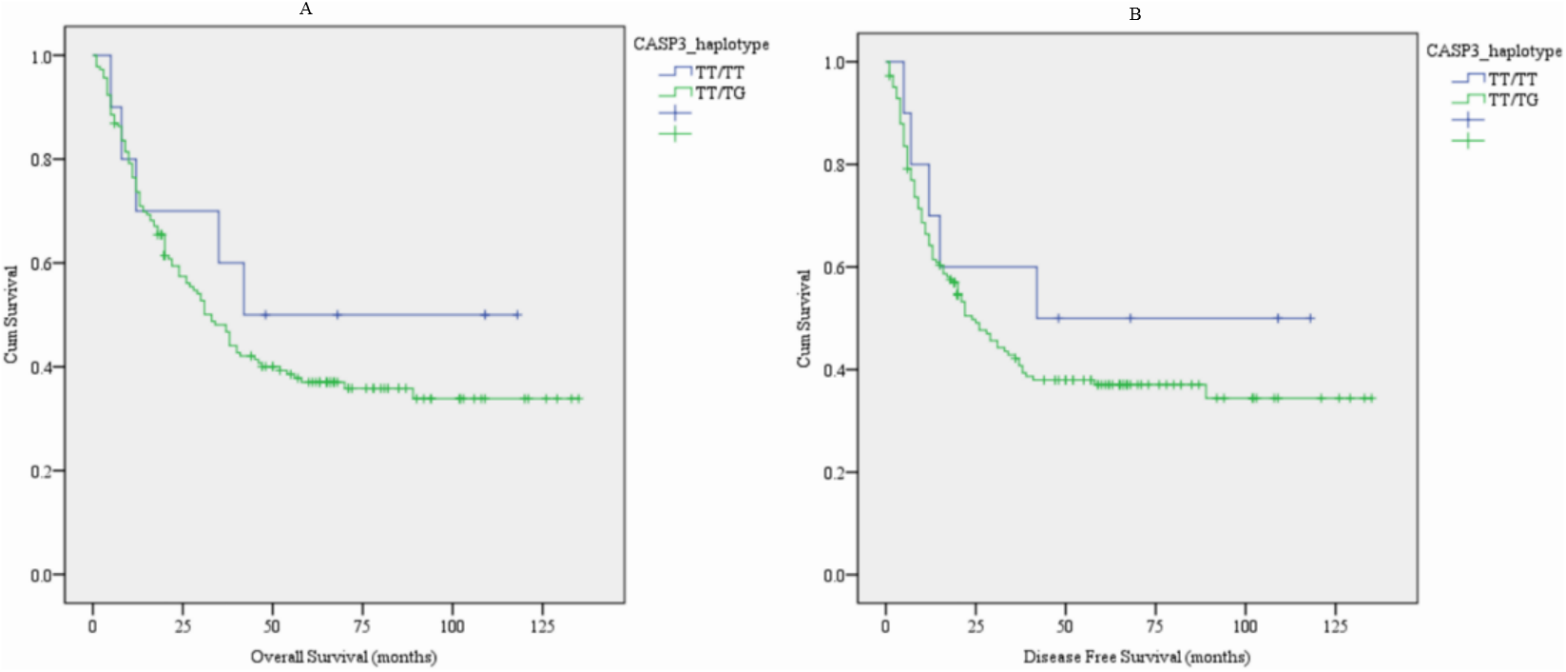
Kaplan-Meier survival curves of *CASP3*_haplotype with clinical outcomes of HCC patients. (A) overall surviva*l* (OS), and (B) disease free survival (DFS).

**Fig 3:**
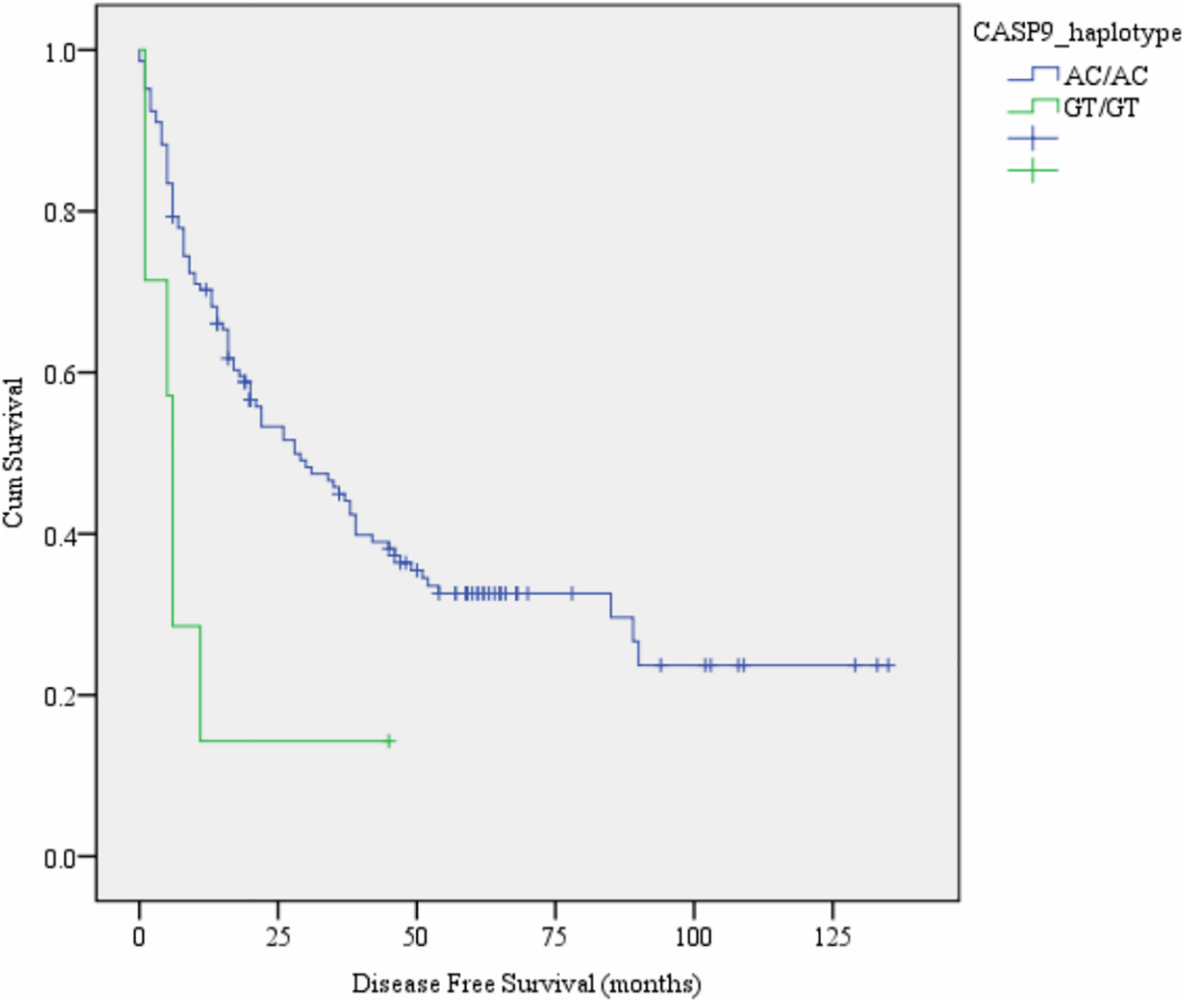
Kaplan-Meier survival curves of *CASP9*_haplotype with disease free survival of HCC patients.

Since two SNPs in both *CASP3* (rs12108497 T>C and rs2705897 T>G) and *CASP9* (rs4645978 A>G and rs4645981 C>T) were selected in the present study, we constructed haplotypes for each of the two genes. We examined the associations of these haplotypes with OS and DFS of HCC patients. The detailed information is shown in Table 4. We attained 7 haplotypes in *CASP3* and 6 haplotypes in *CASP9*. In univariate analysis, *CASP3* haplotype TT/TG was significantly associated with OS (*P* = 0.021, Fig 2A) and DFS (*P* = 0.026, Fig 2B), compared to the common haplotype TT/TT (Table 4). *CASP9* haplotype GT/GT was significantly associated with decreased DFS and showed marginally associated with OS, compared to the common haplotype AC/AC (*P* = 0.012, Fig 3, Table 4).

**Table 4:** Univariate and multivariate Cox regression analysis of haplotypes in HCC patients.

**Haplotype**	**No of patients**	No of events	5-y-survival	MST (95%CI)	OS				DFS			
					Univariate analysis		Multivariate analysis		Univariate analysis		Multivariate analysis	
					Hazard ratio (95% CI)	*P*	Hazard ratio (95% CI)	*p*^a^	Hazard ratio (95% CI)	*P*	Hazard ratio (95% CI)	*p*^a^
***CASP3*_haplotype**												
**TT/TT**	183	108	37	33.0 (25.7–40.4)	1.0		1.0		1.0		1.0	
**CG/CG**	10	5	47	42.0	0.69 (0.28–1.69)	0.416	0.64 (0.26–1.58)	0.333	0.68 (0.28–1.66)	0.395	0.62 (0.25–1.53)	0.299
**CG/TT**	62	43	34	36.0 (14.3–57.7)	1.19 (0.84–1.70)	0.325	1.19 (0.83–1.69)	0.350	1.26 (0.88–1.79)	0.202	1.26 (0.88–1.80)	0.200
**CT/CG**	12	8	36	26.0 (10.7–41.7)	1.08 (0.53–2.21)	0.840	0.98 (0.47–2.02)	0.954	1.00 (0.49–2.05)	0.996	0.90 (0.44–1.86)	0.782
**CT/CT**	8	5	60	72.0(27.4–116.6)	0.80 (0.33–1.96)	0.622	0.97 (0.39–2.41)	0.939	0.78 (0.32–1.92)	0.592	0.97 (0.39–2.42)	0.939
**TT/CT**	61	38	36	38.0 (23.4–52.6)	1.07 (0.74–1.54)	0.735	1.12 (0.77–1.62)	0.562	1.10 (0.76–1.60)	0.607	1.13 (0.78–1.64)	0.512
**TT/TG**	22	18	24	17.0 (8.0–26.0)	1.81 (1.09–2.98)	0.021	1.77 (1.08–2.93)	0.025	1.77 (1.07–2.91)	0.026	1.74 (1.05–2.87)	0.030
***CASP9*_haplotype**												
**AC/AC**	145	93	35	37.0 (19.0–45.0)	1.0		1.0		1.0		1.0	
**AC/GC**	100	61	36	30.0 (15.4–44.6)	1.06 (0.76–1.46)	0.742	1.10 (0.79–1.52)	0.574	1.09 (0.79–1.51)	0.590	1.12 (0.81–1.55)	0.504
**GC/GC**	24	13	42	26.0 (2.9–49.1)	0.89 (0.50–1.60)	0.704	0.87 (0.49–1.57)	0.653	0.88 (0.49–1.58)	0.678	0.83 (0.46–1.51)	0.549
**GC/GT**	19	13	28	33.0 (11.4–54.6)	1.14 (0.64–2.03)	0.666	1.23 (0.68–2.21)	0.498	1.11 (0.62–1.99)	0.717	1.18 (0.66–2.13)	0.571
**GT/AC**	64	37	43	38.0 (19.7–56.3)	0.87 (0.59–1.27)	0.462	0.83 (0.56–1.21)	0.328	0.87 (0.59–1.27)	0.464	0.83 (0.57–1.22)	0.340
**GT/GT**	7	6	23	8.0 (2.9–13.1)	2.19 (0.95–5.02)	0.064	2.35 (0.98–5.64)	0.055	2.91 (1.26–6.71)	0.012	3.16 (1.32–7.57)	0.010

MST, median survival time; CI, confidence interval.

^a^ Adjusted by tumor size and venous invasion.

Similar results were found in multivariate analysis adjusted for tumor size and venous invasion. The *CASP3* haplotype TT/TG presented a negative effect on OS (*P* = 0.025) and DFS (*P* = 0.030) of HCC patients, compared to the common haplotype TT/TT (Table 4). Meanwhile, *CASP9* haplotype GT/GT presented a negative effect on DFS (*P* = 0.010) and suggestively negative effect on OS (*P* = 0.055) of HCC patients, compared to the common haplotype AC/AC (Table 4).

## Discussion

Though many investigations have reported associations of SNPs in CASP genes with several types of cancer, studies of genetic polymorphisms in CASP genes on susceptibility to HCC is few, not to mention relationship between CASP polymorphisms and prognosis of HCC. The aim of our study was to evaluate genetic variants of CASP genes in relation to survival outcomes of HCC patients. To the best of our knowledge, this is the first evidence showing the relationship between genetic polymorphisms of CASP genes and prognosis of HCC patients. Our results revealed that *CASP9* rs4645981 C allele was significantly increased DFS compared with the T allele and only the CT genotype was significantly associated with positive effect on OS, compared with the TT genotype. Moreover, the haplotype TT/TG (constructed by rs12108497 T>C, rs2705897 T>G) in *CASP3* gene was significantly associated with decreased OS and DFS. The haplotype GT/GT in *CASP*9 was only significantly associated with decreased DFS. These findings suggest that the *CASP9* rs4645981 and the haplotype TT/TG in *CASP3 and* GT/GT in *CASP*9 may be useful markers for predicting prognosis of HCC patients.

Failure of apoptosis is a hallmark of human cancers. As an effector CASP, *CASP3* plays an important role in the execution phase of apoptosis, also in the development and progression of cancers [16–17]. Several previous studies have shown associations of *CASP3* polymorphisms on the risk of different types of cancer, including HCC [13, 18–20]. Moreover, studies in several tumor types indicated that the expression levels of *CASP3* have effects on the development and survival of cancers [8, 17, 21]. In our study, the haplotype TT/TG (constructed by rs12108497 T>C, rs2705897 T>G) was significantly associated with decreased OS and DFS in patients with HCC. The findings of previous studies and ours suggest that polymorphisms in *CASP3* may increase risk of development of HCC and lead to poor survival outcome in patients with HCC, through reducing the apoptotic capacity.

CASPs have two distinct but converging pathways for activation, including extrinsic pathway and intrinsic pathway. *CASP9*, an important initiator CASP of the intrinsic pathway, is activated by the release of cytochrome c from mitochondria, activates downstream the effector *CASP3* and *CASP7* [22–23]. Many previous studies have shown that polymorphisms in *CASP9* were associated with various cancer types, especially in the promoter region. For example, Theodoropoulos GE et al. [22] evaluated the association between two SNPs (rs4645978, rs4645981) in the promoter region of *CASP9* and the risk of breast cancer. They found that the rs4645978 G allele was at high risk for breast cancer development and similar results for the rs4645981 T allele, which was significantly associated with increased risk of breast cancer, compared with those harboring the CC genotype. However, Park JY et al. [24] found that *CASP9* rs4645978 polymorphism played a protective role in susceptibility to lung cancer risk and the rs4645981 T allele was at a significantly increased risk of lung cancer compared with those harboring the CC genotype. Moreover, previous studies demonstrated that *CASP9* polymorphisms and expression were associated with prognosis of cancers [25–26]. To our best knowledge, the present study showed the first evidence of association between polymorphisms in *CASP9* and the prognosis of HCC patients.

We acknowledge that there were several limitations in our study. First, the sample size of the present study was relatively small. Therefore, larger sample size and follow-up studies are warranted to confirm our findings. Second, determination of the exact functional influence was not performed in our study. Functional studies on biological mechanisms are needed to investigate in further studies. Third, other treatment information such as whether or not receiving targeted therapy was not collected in our study, except for surgery which is the most important factor for prognosis of patients. Finally, though two clinical and pathologic characteristics showed significant associations with OS and DFS, including tumor size and venous invasion, it is regretful that we failed to collect accurate information of these factors in our study. We only performed multivariate analysis by adjusting these potential prognostic factors. Further studies are essential to evaluate the role of genetic polymorphisms in HCC patients with more complete and comprehensive clinical pathologic characteristics.

In conclusion, our results provide suggestive evidence that *CASP9* and *CASP3* genetic polymorphisms may be independent prognosis markers for HCC patients with surgical resection of tumor. This study is the first evidence showing the relationship between genetic polymorphisms of CASP genes and survival outcomes in HCC patients, more comprehensive studies are needed to confirm our findings and investigate the associations between CASP genetic polymorphisms and prognosis of HCC patients.

## Supporting information

S1 File Association analysis of clinical characteristics, genotypes and haplotypes with OS and DFS of HCC patients. (ZIP)
